# Pectolinarigenin Improves Oxidative Stress and Apoptosis in Mouse NSC-34 Motor Neuron Cell Lines Induced by C9-ALS-Associated Proline–Arginine Dipeptide Repeat Proteins by Enhancing Mitochondrial Fusion Mediated via the SIRT3/OPA1 Axis

**DOI:** 10.3390/antiox12112008

**Published:** 2023-11-16

**Authors:** Ru-Huei Fu

**Affiliations:** 1 Graduate Institute of Biomedical Sciences, China Medical University, Taichung 40402, Taiwan; rhfu@mail.cmu.edu.tw; Tel.: +886-422052121-12486; 2 Ph.D. Program for Aging, China Medical University, Taichung 40402, Taiwan; 3 Translational Medicine Research Center, China Medical University Hospital, Taichung 40447, Taiwan

**Keywords:** amyotrophic lateral sclerosis (ALS), *C9orf72*, proline–arginine dipeptide repeat protein (PR-DPR), pectolinarigenin, ROS, mitochondrial dynamics, apoptosis, OPA1, acetylation, SIRT3

## Abstract

Amyotrophic lateral sclerosis (ALS) is considered a fatal progressive degeneration of motor neurons (MN) caused by oxidative stress and mitochondrial dysfunction. There are currently no treatments available. The most common inherited form of ALS is the *C9orf72* mutation (C9-ALS). The proline–arginine dipeptide repeat protein (PR-DPR) produced by C9-ALS has been confirmed to be a functionally acquired pathogenic factor that can cause increased ROS, mitochondrial defects, and apoptosis in motor neurons. Pectolinarigenin (PLG) from the traditional medicinal herb *Linaria vulgaris* has antioxidant and anti-apoptotic properties. I established a mouse NSC-34 motor neuron cell line model expressing PR-DPR and confirmed the neuroprotective effect of PLG. The results showed that ROS production and apoptosis caused by PR-DPR could be improved by PLG treatment. In terms of mechanism research, PR-DPR inhibited the activity of the mitochondrial fusion proteins OPA1 and mitofusin 2. Conversely, the expression of fission protein fission 1 and dynamin-related protein 1 (DRP1) increased. However, PLG treatment reversed these effects. Furthermore, I found that PLG increased the expression and deacetylation of OPA1. Deacetylation of OPA1 enhances mitochondrial fusion and resistance to apoptosis. Finally, transfection with Sirt3 small interfering RNA abolished the neuroprotective effects of PLG. In summary, the mechanism by which PLG alleviates PR-DPR toxicity is mainly achieved by activating the SIRT3/OPA1 axis to regulate the balance of mitochondrial dynamics. Taken together, the potential of PLG in preclinical studies for C9-ALS drug development deserves further evaluation.

## 1. Introduction

Amyotrophic lateral sclerosis (ALS) is a relatively common rare disease characterized by progressive degeneration of the upper and lower motor neurons. Most ALS cases are sporadic and may be related to environmental pollution, poisons, trauma, and infection. Only approximately 10% of cases are familial ALS [1]. The most prevalent of these is the *C9orf72* (Gene ID: 203228) mutation (C9-ALS) [2]. C9-ALS is a large expansion of the GGGGCC hexanucleotide repeat sequence (HRE) in intron 1. Current research shows that this amplification can downregulate *C9orf72* expression (loss of function), leading to functional defects in immunity, autophagy, actin, and endocytosis. Moreover, the HRE portion produces RNA or protein factors with toxic repeat sequences (gain of function). The interaction of these three effects may result in a toxic gain. HRE sequences are known to generate five dipeptide repeat protein sequences (DPR) in either sense or antisense orientation via the repeat-associated non-ATG translation (RAN) mechanism [3]. Research shows that PR-DPR, a protein with a strong positive charge, is the most toxic. It mainly enters the cell nucleus to form aggregates and affects various physiological functions of cells [4,5].

A previous study showed that C9-ALS causes neuronal mitochondrial damage [6,7]. These damages included (1) Decreased mitochondrial DNA (mtDNA) synthesis [8]. (2) Downregulated expression of genes encoding the electron transport chain [9,10,11,12]. (3) Induced ubiquitination and degradation of ATP5A1 in electron transport chain complex V [13]. (4) Decreased mitochondrial membrane potential (MMP) [14]. (5) Destruction of the mitochondrial inner membrane structure [15]. (6) Loss of mitochondrial calcium homeostasis [16,17,18]. (7) Inhibition of mitophagy activity [19]. In addition, C9-ALS reduced the expression of the mitochondria-protecting antioxidant enzyme NRF2 [14]. In some studies, C9-ALS has been shown to increase p53 phosphorylation, Bax/Bak protein levels, caspase-3 cleavage, and cytochrome c release, thereby promoting apoptosis. In contrast, it inhibits the expression of anti-apoptotic Bcl-xL mRNA [11,20]. All these defects reduce ATP synthesis, oxidative stress, and apoptosis in motor neurons [21]. DPR has also been shown to affect mitochondrial function, particularly those DPR containing arginine. Shorter and less active mitochondria have been observed in the cortical neurons of glycine-agarine (GR)-DPR mice [13].

The homeostasis of mitochondrial dynamics is essential for maintaining the quality and shape of mitochondria, which is accompanied by fission and fusion [22]. Its defects can affect the survival of cells, particularly neurons. Mechanistically, optic atrophy 1 (OPA1, Gene ID: 4976) and mitofusin 1 (Gene ID: 55669) and 2 (Gene ID: 9927) (MFN1 and MFN2) mediate mitochondrial fusion. Conversely, dynamin-related protein 1 (DRP1, Gene ID: 10059) and fission 1 (Fis1, Gene ID: 51024) are involved in the regulation of mitochondrial fission [23]. OPA1 is a mitochondrial guanosine triphosphatase (GTPase) that is widely distributed in the inner membrane of mitochondrial cristae. Its physiological and functional roles include regulating mitochondrial fusion, controlling mitochondrial cristae structure, maintaining mitochondrial integrity, and inhibiting apoptosis-related cytochrome c release [24]. Mitochondrial disruption, respiratory complex I inactivation, and reduced ATP production have been observed in neural cells derived from OPA1-mutated induced pluripotent stem cells [25]. Increased DRP1 expression and suppression of OPA1 activity were observed in the cortical neurons of GR-DPR mice. Regulation of OPA1 activity is affected by acetylation, proteolysis, and SUMOylation [26,27]. Acetylation modification of OPA1 at lysine 926 and 931 reduces the GTPase activity of the protein. Mitochondrial deacetylase SIRT3 can deacetylize OPA1 and increase its GTPase activity [28].

NAD-dependent deacetylase SIRT3 (Gene ID: 23410) is a member of the mammalian sirtuin protein family and is located in the mitochondrial soluble matrix [29]. Research has shown that SIRT3 plays a central role in the regulation of aging, mitochondrial activity, autophagy, and oxidative stress [30]. SIRT3 can inhibit mitochondrial dysfunction, reduce the production of ROS, induce mitophagy, and inhibit apoptosis; therefore, it can be used to treat neurodegenerative diseases. [31].

**Figure 1:**
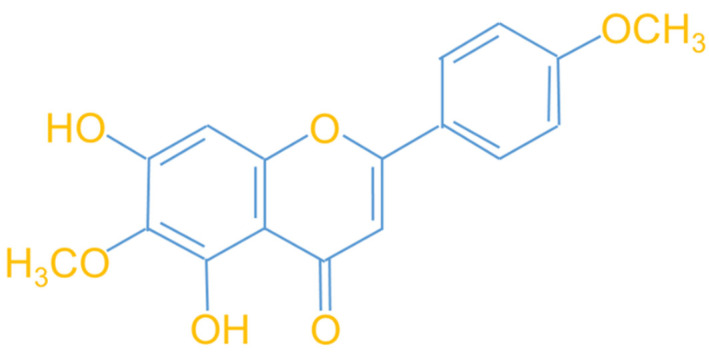
Molecular structure diagram of the flavonoid compound pectolinarigenin (PLG) derived from *Linaria vulgaris*.

The natural flavonoid pectolinarigenin (PLG, Figure 1) was derived from *Linaria vulgaris*, a traditional medicinal herb. It has been shown to possess anti-oxidative [32], anti-inflammatory [33], neuroprotective [34], and anti-cancer properties. [35]. However, the therapeutic utility of PLG in C9-ALS has not yet been evaluated. Mouse NSC-34 motor neuron cell lines are commonly used to construct pharmacological and transgenic ALS in vitro models. It can be used to evaluate the effectiveness and mechanism of ALS treatment strategies [36]. In this study, I established an NSC-34 motor neuron cell line expressing PR_50_. I found that PR_50_ overexpression increases intracellular ROS production and causes mitochondrial dynamics defects that cause apoptosis. Treatment with PLG ameliorates these damages. This ability may be achieved in part by enhancing the SIRT3-OPA1 axis to maintain the dynamic balance of mitochondria.

## 2. Materials and Methods

### 2.1. Experimental Reagents, NSC-34 Cells and Culture Media

Synthetic pectolinarigenin (PLG) and other chemicals were obtained from Sigma-Aldrich (St. Louis, MO, USA) unless otherwise indicated. PLG was prepared into 10 mM stock solution. Culture media and various supplements were purchased from Gibco (Thermo Fisher Scientific, Waltham, MA, USA). The NSC-34 cell line (mouse motor neuron-like hybrid cells) provided by Professor Shinn-Zong Lin (Tzu Chi University, Hualien, Taiwan) was cultured in Dulbecco’s modified Eagle medium (DMEM) supplemented with 10% fetal bovine serum (FBS) and 1% penicillin (100 units/mL)/streptomycin (100 μg/mL) in an incubator with a humidified atmosphere of 5% CO_2_ at 37 °C.

### 2.2. PR_50_-Expressing Plasmid Construction and Transfection of NSC-34 Cells

DNA fragments of PR_50_ were generated by gene synthesis (Genewiz, South Plainfield, NJ, USA). The fragment was digested by the restriction enzymes *Xho I* and *Hind III* and inserted into the pcDNA 3.1/myc-His vector (Invitrogen, ThermoFisher Scientific, Carlsbad, CA, USA). Finally, according to the manufacturer’s instructions, Lipofectamine 2000 reagent (Invitrogen) was used to transfect the plasmid into the NSC-34 cell line. Transfected cells were selected and maintained using G418 (1.5 mg/mL).

### 2.3. Immunofluorescence Staining of NSC34 Cells

NSC-34 cells grown on coverslips were washed and treated with paraformaldehyde (4%) and Triton X-100 (0.2%). The blocking solution and anti-Myc antibody (Cell Signaling Technology, Beverly, MA, USA) were then added to the coverslip for reaction overnight at 4 °C. The next day, cells were washed and incubated with Alexa Fluor 488-conjugated goat anti-rabbit IgG secondary antibody (Invitrogen) for 1 h at room temperature. The washed cells were then stained with DAPI for nuclei. Fluorescent signals were detected using a Zeiss Axio Imager A1 fluorescence microscope (Carl Zeiss MicroImaging GmbH, Göttingen, Germany).

### 2.4. Mitochondrial Membrane Potential Determination of NSC-34 Cells

First, I replaced the cultured NSC-34 cells with fresh medium and added 3,3’-dihexyloxycarbocyanine iodide (DiOC6) (1 μM) for staining. After 30 min, an Axio Observer inverted fluorescence microscope (Carl Zeiss MicroImaging GmbH, Göttingen, Germany) was used to detect the green fluorescence associated with the MMP intensity, and the fluorescence intensity was quantified using ImageJ software (version 1.53, National Institutes of Health).

### 2.5. TUNEL Assay of NSC-34 Cells

After the NSC-34 cells on the coverslip were fixed and permeabilized, the Click-iT™ Plus TUNEL detection kit (Invitrogen) was used according to the manufacturer’s instructions to detect in situ cell apoptosis using fluorescence microscopy and calculate the proportion of apoptotic cells.

### 2.6. Flow Cytometry of Annexin V (FITC) and Propidium Iodide Staining of NSC-34 Cells

NSC-34 cells were subjected to apoptosis detection using the FITC Annexin-V Apoptosis Detection Kit I (BD Biosciences Pharmingen, San Diego, CA, USA) according to the manufacturer’s instructions. Cell staining results for FITC-labeled Annexin V and propidium iodide were determined using a BD LSRII flow cytometer (Becton Dickinson, Heidelberg, Germany). The instrument sets a collection gate limit of 10,000 events per sample.

### 2.7. Western Blotting Analysis of Protein Expression in NSC-34 Cells

NSC-34 cells were washed using fully formulated RIPA buffer (ThermoFisher Scientific) to collect cell lysates and determine total protein concentration using the Coomassie Plus Protein Assay Kit (Pierce, Rockford, IL, USA). I ran 50 µg of cell lysate through SDS-PAGE (7.5–12.5%) and transferred it to a PVDF membrane (Merck Millipore, Carrigtwohill Co. Cork, Ireland). Next, the membrane was incubated with specific antibodies overnight. The next day, I washed the membrane and added horseradish peroxidase (HRP)-conjugated secondary antibody for reaction at room temperature for 1 h. Finally, the location and levels of specific proteins in the membrane were determined using an Amersham enhanced chemiluminescence kit (Amersham Biosciences, Piscataway, NJ, USA) and a BioSpec-trum imaging system (UVP, Upland, CA, USA). Caspase 3, poly ADP ribose polymerase (PARP), GAPDH, OPA1, MFN2, Fis1, DRP1, SIRT3, and acetylated lysine antibodies were obtained from Cell Signaling Technology. HRP-conjugated goat anti-mouse and goat anti-rabbit secondary antibodies were purchased from PerkinElmer, Inc. (Boston, MA, USA).

### 2.8. Viability Analysis of NSC-34 Cells

NSC-34 cells transfected with PR_50_ or empty vector were incubated in 96-well culture plates (4 × 10^3^cell/pre well) to reach 70% confluence. Next, serially diluted PLG was added and treated for 24 h. CellTiter-Blue^®^ Reagent (Promega, Madison, WI, USA) was used according to the manufacturer’s instructions. Finally, the survival ratio of cells was quantified using the SpectraMax M2 system (Molecular Devices, Silicon Valley, CA, USA).

### 2.9. Quantitative Analysis of ROS in NSC-34 Cells

NSC-34 cells were cultured on a black 96-well plate, and then fresh medium was placed and 2′,7′-dichlorodihydrofluorescein diacetate (H2DCFDA, 25 μM) was added. After incubation at 37 °C in the dark for 30 min, the cells were washed and the fluorescence intensity was measured every 15 min with a spectrophotometer for 150 min. 

### 2.10. Citrate Synthase Activity Assay of Mitochondrial in NSC-34 Cells

NSC-34 cells were washed and lysed using low-strength complete formulation RIPA buffer (Thermo Fisher Scientific, Waltham, MA, USA) to obtain whole-cell extracts. Next, I analyzed 50 μg of the extract using a citrate synthase assay kit (Sigma-Aldrich, St. Louis, MO, USA) according to the manufacturer’s instructions and measured the values with a spectrophotometer.

### 2.11. Immunoprecipitation (IP) Assay of NSC-34 Cells

NSC-34 cells were washed and lysed using low-strength complete formulation RIPA buffer (Thermo Fisher Scientific, Waltham, MA, USA) to obtain whole-cell extracts. Next, cell extracts were cleared using Protein A-Sepharose beads and incubated overnight at 10 °C with anti-OPA1 antibody or normal rabbit immunoglobulin G (Cell Signaling Technology). The next day, the cell extract/OPA1 antibody was added to Protein A-Sepharose beads (0.1 g/L) and incubated at 10 °C for 4 h. Finally, the immunoprecipitate-bound Protein A-Sepharose beads were collected by centrifugation, and the acetylation level of OPA1 was determined by Western blotting using an acetylated lysine antibody.

### 2.12. Transient Transfection of Small Interfering RNAs of NSC-34 Cells

NSC-34 cells (2.0 × 10^5^) were seeded on a 6-well culture plate. When 70% confluence was reached, transfection with sirt3 or non-targeting control small interfering RNA (siRNA; 75 nM) was performed for 16 h. Transfection was implemented using Lipofectamine 2000 transfection reagent (Invitrogen) according to the manufacturer’s instructions. siRNA targeting mouse sirt3 and control siRNA were purchased from Thermo Fisher Scientific.

### 2.13. Statistical Analysis of the Experimental Results

Each experiment was repeated at least three times. Statistical analysis of data was performed using SAS software (version 9.4, SAS Institute Inc., Cary, NC, USA). Data are presented as mean ± standard deviation (SD). Statistical significance was determined using one-way ANOVA and Tukey’s post hoc test. A *p* value < 0.05 was considered significant.

## 3. Results

### 3.1. PR_50_ Expression Causes Apoptosis of Mouse NSC-34 Motor Neuron Cells

**Figure 2:**
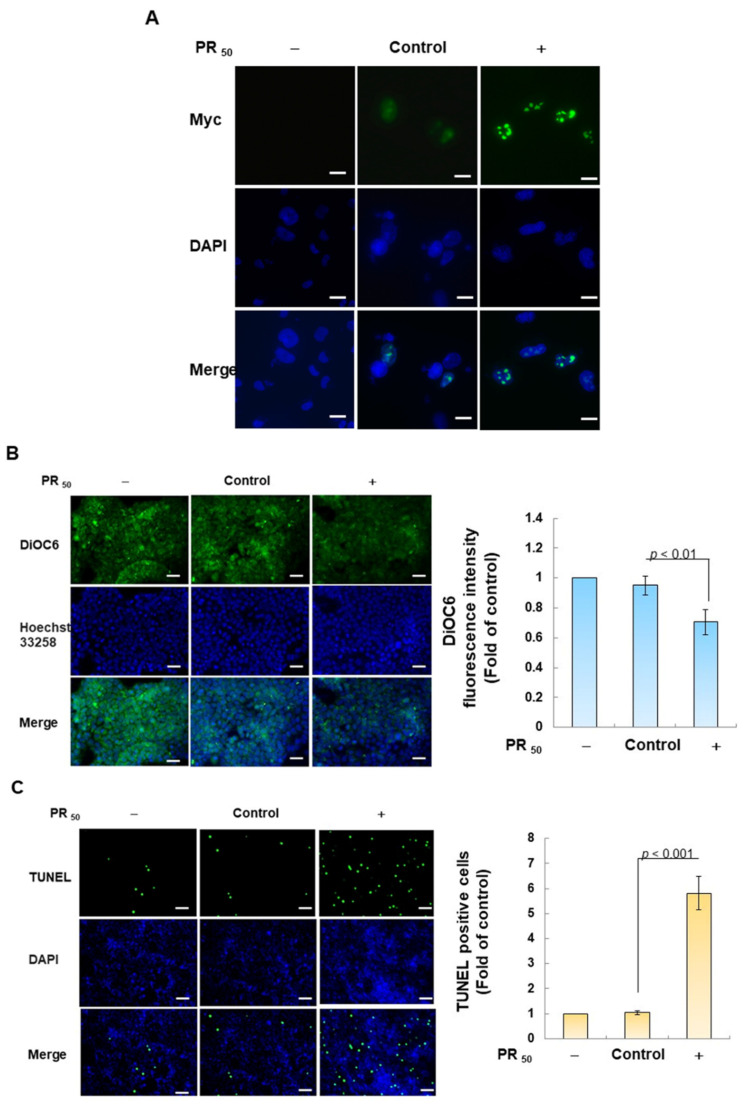

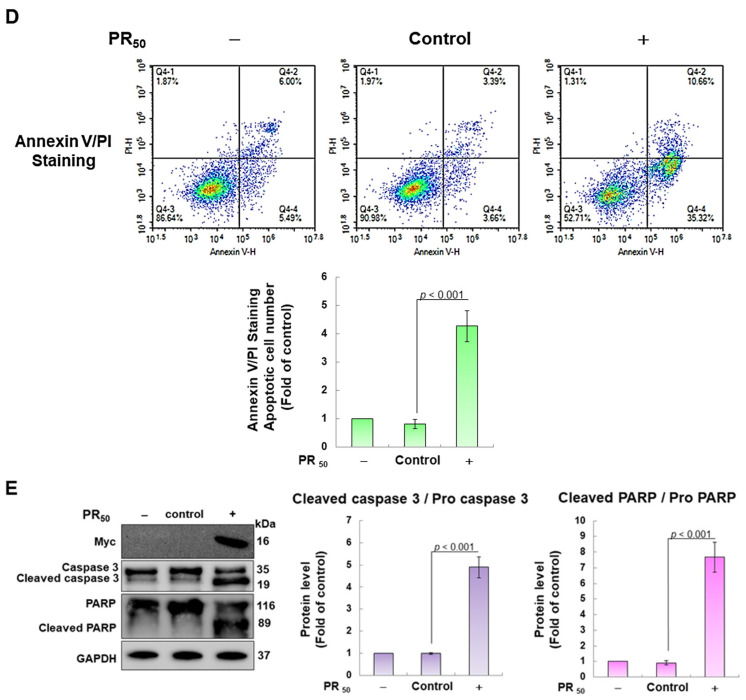
PR_50_ expression causes apoptosis of mouse NSC-34 motor neuron cells. PR_50_ plasmids were transfected into NSC-34 cells for 24 h. (**A**) immunofluorescent staining using anti-myc-tag antibody (green). The position of the nucleus was confirmed by DAPI staining (blue). Microscope magnification is 400x. PR_50_ mainly forms punctates in the nucleus. (**B**) DiOC6 staining and fluorescence microscopy were used to detect changes in mitochondrial membrane potential (MMP). Microscope magnification is 100x. Quantitative values were obtained using ImageJ software (version 1.53). (**C**) the proportion of cells with chromatin fragmentation was assessed by fluorescence microscopy using the TUNEL assay. Microscope magnification is 100x. (**D**) cells co-stained with FITC-conjugated annexin V and propidium iodide (PI) were analyzed by flow cytometry to quantify the proportion of apoptotic cell populations. (**E**) the expression of apoptosis-related proteins was determined by Western blotting. GAPDH was used to normalize the total protein loading in each group. Quantitative values were obtained using ImageJ software (version 1.53). The results of Western blotting show the presence of the active form of the proteins since they are activated by proteolysis.

First, I established a mouse NSC-34 motor neuron cell line model with temporary PR_50_ expression. Immunofluorescence staining showed that PR_50_ mainly formed punctates in the nucleus (Figure 2A). Next, I evaluated the effect of PR_50_ on the induction of apoptosis. I used DiOC6 staining to observe changes in the mitochondrial membrane potential (MMP) of cells. The results showed that PR_50_ expression caused a loss of MMP compared with the empty vector (*p* < 0.01, Figure 2B). In observing the fragmentation of chromatin DNA in situ, the TUNEL assay revealed that PR_50_ expression significantly caused chromosomal DNA fragmentation compared with the empty vector group (*p* < 0.001, Figure 2C). On the quantitative apoptotic cell population, flow cytometry analysis of cells co-stained with annexin V and propidium iodide indicated that the PR_50_ expression group significantly promoted cell apoptosis compared with the empty vector group (*p* < 0.001, Figure 2D). Finally, I assessed the activation of apoptosis-related core proteins using Western blotting. The results showed that PR_50_ expression caused a significant increase in the ratios of cleaved caspase 3/pro caspase 3 (*p* < 0.001) and cleaved PARP/pro PARP (*p* < 0.001) (Figure 2E). According to the above results, PR_50_ expression can induce mitochondrial damage and apoptosis in NSC-34 motor neuron cells. This is consistent with the results observed in the motor neurons of patients and mice with C9-ALS.

### 3.2. Pectolinarigenin (PLG) Treatment Improves the Viability of PR_50_-Expressing NSC-34 Cells and Prevents Apoptosis

**Figure 3:**
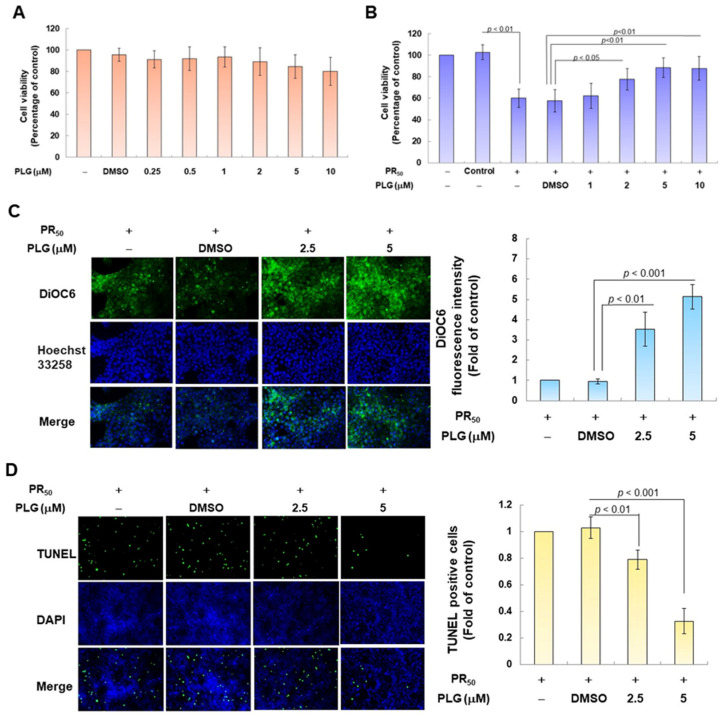

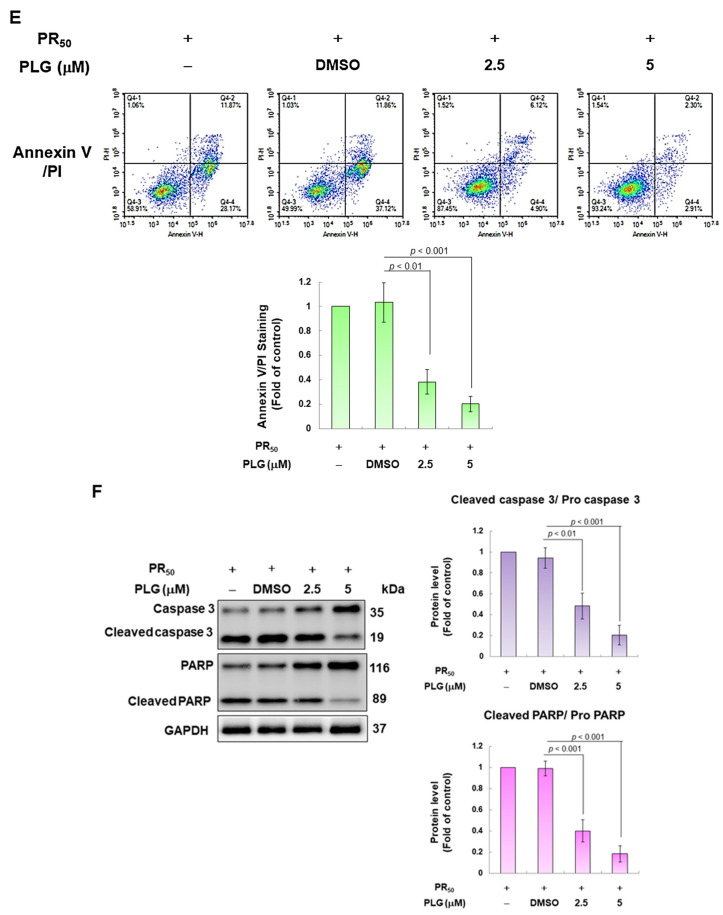
PLG treatment can alleviate PR_50_-induced apoptosis of NSC-34 motor neuron cells. (**A**) NSC-34 cells were treated with different concentrations of PLG for 24 h, and cell viability was determined using the CellTiter Blue Cell Viability Assay. (**B**) PR_50_-expressing NSC-34 cells were treated with different concentrations of PLG for 24 h, and cell viability was measured. (**C**–**F**) PR_50_-expressing NSC-34 cells were treated with 2.5 and 5 μM PLG for 24 h. (**C**) DiOC6 probe was used to detect changes in mitochondrial membrane potential (MMP) through fluorescence microscopy. Microscope magnification is 100x. The signal intensity was quantified using ImageJ software (version 1.53). (**D**) the number of cells with chromatin fragmentation was counted by fluorescence microscopy using the TUNEL assay. Microscope magnification is 100x. (**E**) FITC-conjugated Annexin V- and propidium iodide (PI) co-stained cells were used to count the number of apoptotic cell populations using flow cytometry. (**F**) the expression of apoptosis-related proteins was determined by Western blotting. GAPDH was used to normalize the total protein loading in each group. Quantitative values were obtained using ImageJ software (version 1.53). The results of Western blotting show the presence of the active form of the proteins since they are activated by proteolysis.

Because pectolinarigenin (PLG) is known to have antioxidant and neuroprotective properties, I aimed to evaluate its effect on improving apoptosis induced by PR_50_ expression in NSC-34 motor neurons. First, I confirmed the appropriate concentration for PLG treatment. Cell viability analysis showed that treatment with PLG at concentrations lower than 10 μM did not affect the survival of NSC-34 cells (Figure 3A). In addition, compared with the empty plasmid group, PR_50_ expression significantly inhibited cell survival (*p* < 0.001, Figure 3B), which confirmed that PR_50_ can cause cytotoxicity. I further found that PLG treatment dose-dependently increased the viability of PR_50_-expressing cells at concentrations above 1 μM compared with the PR_50_/DMSO group (5 μM group, *p* < 0.01, Figure 3B). However, there was no significant difference in the effect on cell survival rate when treated with 10 μM PLG compared with 5 μM. Therefore, we selected PLG concentrations of 2.5 and 5 μM for analysis in subsequent experiments.

In the fluorescence analysis of the DiOC6 probe, compared with the PR_50_/DMSO group, PLG restored the mitochondrial membrane potential (MMP) of PR_50_-expressing cells (5 μM group, *p* < 0.001, Figure 3C). TUNEL assay analysis revealed that compared with the PR_50_/DMSO group, the number of positive cells in the PR_50_/PLG group decreased (5 μM group, *p* < 0.001, Figure 3D). Furthermore, flow cytometry was used to analyze cells co-stained with annexin V and propidium iodide. The results indicated that the total number of early and late apoptotic cells in the PR_50_/PLG group was significantly reduced compared with that in the PR_50_/DMSO group (5 μM group, *p* < 0.001, Figure 3E). Finally, Western blot analysis displayed that compared with the PR_50_/DMSO group, expressions of the cleaved apoptotic core proteins, cleaved caspase 3/pro caspase 3 (5 μM, *p* < 0.001) and cleaved PARP/pro PARP (5 μM, *p* < 0.001), were lower in the PR_50_/PLG group than in the PR_50_/DMSO group (Figure 3F). According to the above results, PLG treatment can significantly reduce the apoptosis of NSC-34 motor neuron cells caused by PR_50_ expression.

### 3.3. PLG Inhibits the Enhancement of ROS Production and Mitochondrial Fission in NSC-34 Cells Caused by PR_50_

**Figure 4:**
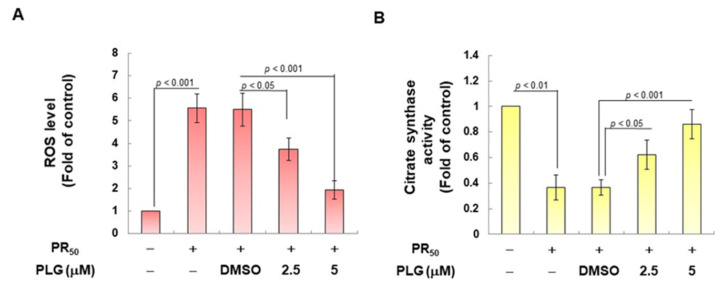

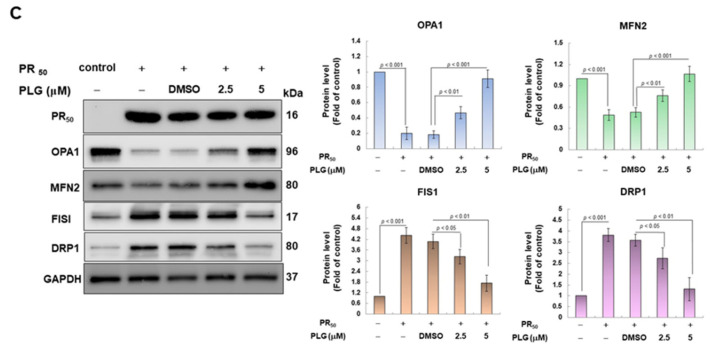
PLG treatment reduced cellular ROS production and increased mitochondrial fusion in PR_50_-expressing NSC-34 cells. PR_50_-expressing NSC-34 cells were treated with PLG for 24 h. (**A**) the H2DCFDA probe was used to measure the ROS level in each group. (**B**) citrate synthase activity assay was used to determine intracellular mitochondrial activity in each group. (**C**) expression of fusion (OPA1 and MFN2) and fission (Fis1 and DRP1)-related proteins on mitochondrial dynamics was assessed using Western blotting. GAPDH was used as the control for total protein loading in each group. The signal intensity was quantitatively analyzed using ImageJ software (version 1.53).

On the basis of the aforementioned results showing that PLG can reduce motor neuron apoptosis caused by PR_50_, I explored the possible mechanism. Because studies have shown that C9-ALS can cause loss of mitochondrial function, including abnormalities in mitochondrial dynamics, I wanted to clarify whether the neuroprotective effects of PLG are accompanied by this pathway. First, H2DCFDA probe analysis showed that PR_50_ expression increased ROS levels in NSC-34 cells (*p* < 0.001, Figure 4A). This reveals that PR_50_ can damage mitochondria and cellular antioxidant mechanisms, thereby leading to an increase in ROS levels. Next, I compared the PR_50_/PLG group with the PR_50_/DMSO group, and the results indicated that PLG could reduce ROS production (5 μM group, *p* < 0.001, Figure 4A). I also evaluated the effect of PLG on the mitochondrial activity of PR_50_-expressing NSC-34 cells by detecting mitochondrial citrate synthase activity. As shown in Figure 4B, PR_50_ expression significantly diminished the mitochondrial activity of NSC-34 cells (*p* < 0.01). However, compared with the PR_50_/DMSO group, the data showed that PLG restored the mitochondrial activity of PR_50_-expressing NSC-34 cells (5 μM group, *p* < 0.001, Figure 4B). I also wanted to determine whether the restoration of mitochondrial activity is related to the promotion of fusion in mitochondrial dynamics. Western blotting revealed that PR_50_ inhibited the expression of the mitochondrial fusion proteins OPA1 (*p* < 0.001) and MFN2 (*p* < 0.001) in NSC-34 cells and promoted the expression of the fission proteins Fis1 (*p* < 0.001) and DRP1 (*p* < 0.001) (Figure 4C). However, PLG treatment reversed the effects of PR_50_ on the expression of these proteins. Compared with the PR_50_/DMSO group, PLG augmented the expression of OPA1 (5 μM group, *p* < 0.001) and MFN2 (5 μM group, *p* < 0.001) in PR_50_-expressing NSC-34 cells (Figure 4C). The expressions of Fis1 (5 μM group, *p* < 0.01) and DRP1 (5 μM group, *p* < 0.01) were inhibited (Figure 4C). These results indicate that ROS production and mitochondrial dynamics defects caused by PR_50_ expression can be improved by PLG treatment.

### 3.4. PR_50_-Induced Acetylation of OPA1 Is Inhibited by PLG

**Figure 5:**
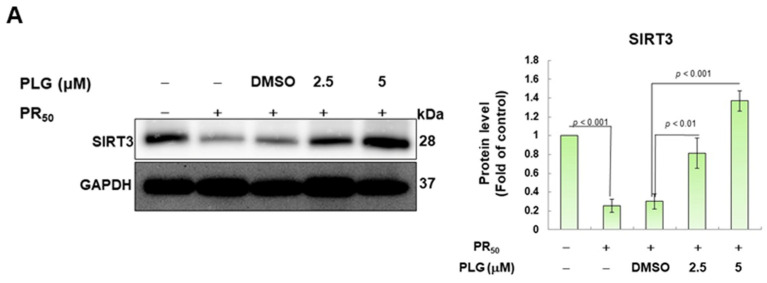

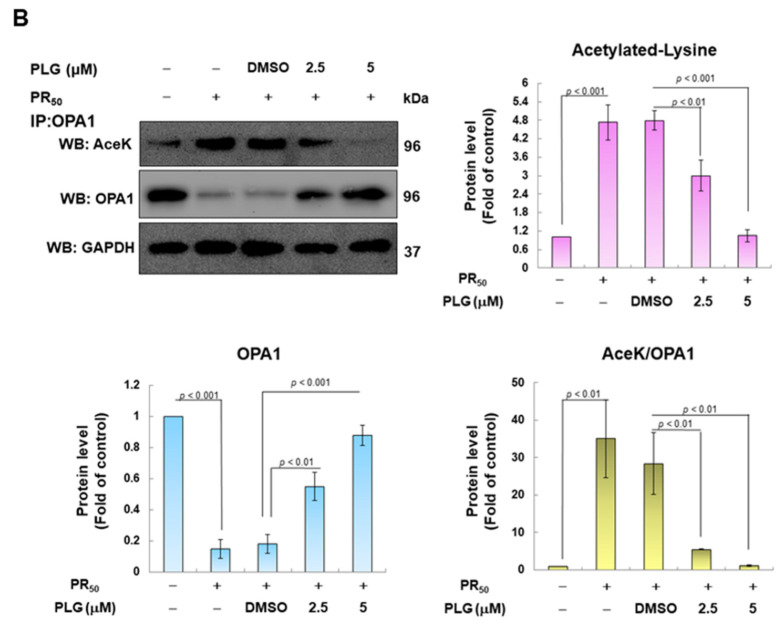
PLG enhances SIRT3 expression and OPA1 deacetylation in PR_50_-expressing NSC-34 cells. PR_50_-expressing NSC-34 cells were treated with PLG for 24 h. (**A**) Western blotting was used to analyze expression. (**B**) determination of the degree of OPA1 acetylation using immunoprecipitation with an OPA1 antibody and Western blotting with a lysine acetylation-specific antibody. GAPDH was used to normalize the loading of all protein extracts from each group. The degree of OPA1 acetylation was corrected by normalizing OPA1 expression levels. The signal intensity was quantified using ImageJ software (version 1.53).

SIRT3 is a deacetylase that broadly regulates mitochondrial homeostasis in vivo, including directing mitochondrial dynamics toward fusion. Western blotting showed that PR_50_ expression lessened SIRT3 expression in NSC-34 cells (*p* < 0.001, Figure 5A). However, compared with the PR_50_/DMSO group, PLG treatment increased SIRT3 levels in PR_50_-expressing cells (5μM group, *p* < 0.001, Figure 5A). Studies have presented that acetylation inhibits the activity of OPA1 and leads to a diminution in mitochondrial fusion ability. Therefore, I evaluated the effect of PLG on the acetylation of OPA1. I immunoprecipitated protein extracts from PR_50_-expressing NSC-34 cells using OPA1 antibodies and then used antibodies against lysine residue acetylation to detect the degree of OPA1 acetylation. The results are shown in Figure 5B. After normalizing the expression of OPA1 in each group compared with that in the empty vector group, PR_50_ expression significantly enhanced the acetylation of OPA1 (*p* < 0.01). Compared with the PR_50_/DMSO group, PLG inhibited the acetylation of OPA1 in PR_50_-expressing NSC-34 cells (5μM group, *p* < 0.01).

### 3.5. SIRT3 Downregulation Abolishes the Ability of PLG to Enhance Mitochondrial Fusion and Resist Apoptosis in PR_50_-Expressing NSC-34 Cells

**Figure 6:**
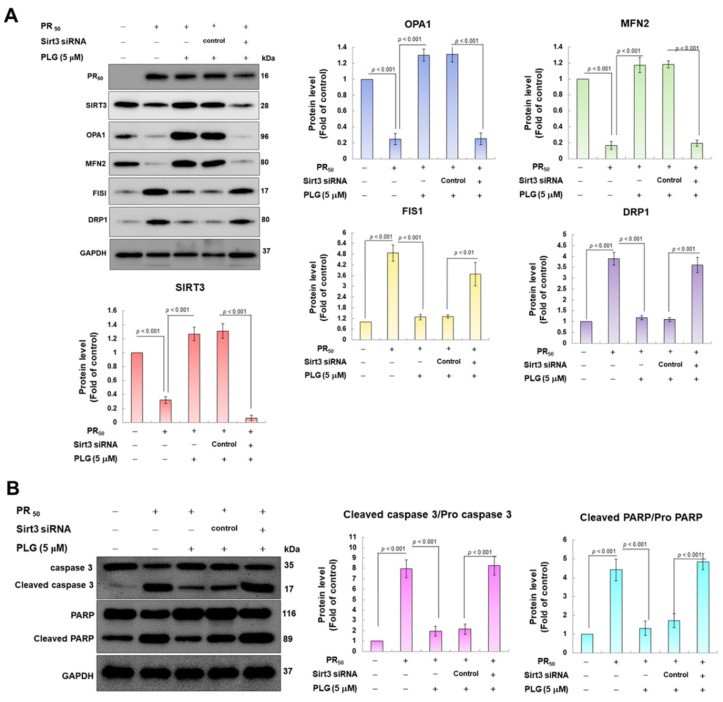
Downregulation of SIRT3 expression eliminates the facilitation of mitochondrial dynamics fusion and the anti-apoptotic activities of PLG in PR_50_-expressing NSC-34 cells. Control or Sirt3 siRNA was delivered to PR_50_-expressing NSC34 cells for 16 h. This was followed by PLG (5 μM) treatment for 24 h. Finally, Western blotting was used to detect the expression of mitochondrial dynamics-related proteins (**A**) and the activity of the apoptotic core protein (**B**). GAPDH served as an internal control for loading total proteins in each group. The signal intensity was quantified using ImageJ software (version 1.53).

Because SIRT3 is the major deacetylase of OPA1, I wondered whether SIRT3 was the major upstream event for the neuroprotective effect of PLG on PR_50_-expressing motor neuron cells. I used siRNA to inhibit SIRT3 expression in PR_50_-expressing cells. Western blotting showed that compared with the control siRNA group, Sirt3 sRNAi inhibited 95% SIRT3 expression in PR_50_-expressing cells (*p* < 0.001, Figure 6A). In the analysis of protein expression related to mitochondrial dynamics, I found that the downregulation of SIRT3 abolished the ability of PLG to promote mitochondrial fusion-related proteins and inhibit fission-related white matter in PR_50_-expressing NSC-34 cells (Figure 6B). Similarly, SIRT3 downregulation also hinders the ability of PLG to prevent expression of the PR_50_-activated cleaved apoptotic core proteins (Figure 6B). These data may demonstrate that PLG reverses mitochondrial dynamics defects and apoptosis induction in PR_50_-expressing NSC-34 cells by regulating SIRT3 activity.

## 4. Discussion

ALS is a fatal degenerative disease of the motor neurons. Although riluzole and edaravone have been approved for the clinical treatment of ALS, their effects are extremely limited. Therefore, it is urgent to develop new therapeutic drugs [1]. C9-ALS accounts for the highest proportion of familial ALS and is present in some patients with sporadic ALS. Therefore, it is a suitable entry point for the development of new drugs for C9-ALS [37]. One of the cytopathological features of C9-ALS is the toxic PR-DPR that occurs via the RAN mechanism. PR-DPR mainly exists in the nucleus and damages various physiological functions of the nucleus, including gene transcription/modification and nucleocytoplasmic transport [38,39]. However, the effects of PR-DPR on many physiological functions outside the nucleus have also been widely reported [40,41,42]. Among them, it has been confirmed that PR-DPR causes mitochondrial function defects, leads to the production of ROS, and finally induces apoptosis [5,43]. Here, I established a mouse NSC-34 motor neuron cell line model expressing PR-DPR (PR50) and used it for drug screening and research on its mechanism of action. In this model, I observed that PR50 expression caused significant damage and apoptosis in NSC-34 cells and increased the production of intracellular ROS. This result is similar to that observed in induced pluripotent stem cell lines producing motor neurons in C9-ALS patients and mouse models [6,44]. Therefore, this model can be used to evaluate the efficacy of C9-ALS drugs and to explore related mechanisms.

Pectolinarigenin (PLG) is a flavonoid compound derived from the traditional medicinal herb *Linaria vulgaris*. It has antioxidant, anti-inflammatory, neuroprotective, and mitochondrial protection functions [45,46,47]. Wu and Liang’s research showed that PLG can improve functional recovery after spinal cord injury in rats and lessen neuronal damage and apoptosis. In addition, PLG prevents the activation of caspase-3, caspase-9, and PARP and decreases the Bax:Bcl2 ratio [48]. Li et al. found that PLG alleviated oxidative stress and cell apoptosis induced by liver ischemia/reperfusion by activating the PI3K/AKT/Nrf2 signaling pathway [46]. Here, I confirmed that PLG also has a significant alleviating effect on ROS production and apoptosis in NSC-34 cells induced by PR-DPR.

The maintenance of mitochondrial morphology, quality, and function requires the balance of mitochondrial dynamics through the fine regulation of fission and fusion processes. Disruption of this homeostasis is associated with neuronal loss in various neurodegenerative diseases such as ALS [49]. Core regulators of mitochondrial fusion include OPA1 and MFN [50]. The initiating factor of the mitochondrial outer membrane fusion process is MFN1/MFN2, whereas the fusion of the mitochondrial inner membrane is mainly related to OPA1. Conversely, DRP1 and Fis 1 promote mitochondrial fission [51]. The accumulation of the ALS-related SOD1^G93A^ mutant protein in mitochondria will cause the downregulation of OPA1, resulting in multiple mitochondrial defects, including ultrastructural disorder, fusion inhibition, maturation obstruction, and cristae swelling, ultimately leading to the loss of mitochondrial membrane potential, reduced respiratory capacity, and increased production of ROS [52]. In Drosophila models, C9-ALS-associated GR-DPR can enter mitochondria and interact with components of the mitochondrial contact site and cristae organizing system (MICOS) and OPA1, altering MICOS dynamics and subunit interactions. This impairs the mitochondrial inner membrane structure, ion homeostasis, mitochondrial metabolism, and energy production [15]. ALS-linked CHCHD10 and TDP-43 mutations disrupt mitochondrial OPA1–mitofilin complex formation, thereby impairing mitochondrial fusion and respiration [53]. My study showed that PR-DPR promotes the upregulation of DRP1 and Fis 1 and inhibits the expression of OPA1 and MFN, leading to an imbalance in mitochondrial dynamics. Finally, it causes cellular oxidative stress and apoptosis. Treatment with PLG can reverse this damage.

The activity of the mitochondrial fusion core protein OPA1 is regulated by deacetylation, proteolysis, and SUMOylation [26,27]. The most important regulatory factor is SIRT3-induced deacetylation [54]. SIRT3 is a mitochondrial NAD-dependent deacetylase involved in the tricarboxylic acid cycle, energy production, and oxidative stress [30]. SIRT3 functions by regulating the level of mitochondrial DNA repair activity, resisting oxidative stress, avoiding cell apoptosis, and maintaining mitochondrial metabolic homeostasis and energy production. It can prevent or alleviate mitochondrial dysfunction and has neuroprotective effects [31]. Modulation of SIRT3 activity now represents a powerful strategy for treating neurodegenerative diseases [55]. A previous study showed that increasing NAD^+^ levels upregulates SIRT3 activity [56]. SIRT3 is usually a SUMOylated protein whose deacetylase activity is temporarily inhibited. Under the stimulation of external factors, the upstream regulatory molecule Sentrin-specific protease 1 (SENP1) de-SUMOylates and activates SIRT3, promoting the deacetylation of downstream target mitochondrial proteins [57]. Mitochondrial defects caused by hyperacetylation of mitochondrial proteins have been found in spinal motor neurons differentiated from induced pluripotent stem cells from patients with familial and sporadic ALS. Activating SIRT3 using nicotinamide or small molecule initiators reverses these defects and corrects a range of ALS-related phenotypes [58]. In SOD1^G93A^ transgenic mice and primary cortical neuronal cells, SIRT3 protects against mitochondrial fragmentation and neuronal cell death in mutant SOD1^G93A^ [59]. In Sirt3 transgenic mice, SIRT3 overexpression increases NADPH levels and protects against oxidative stress-induced cell death [60]. In this study, I found that PLG can enhance the expression of SIRT3, cause OPA1 deacetylation, and finally promote mitochondrial fusion and improve PR_50_-induced apoptosis. Downregulation of SIRT3 abolished this ability of PLG. This confirms activation of the SIRT3–OPA1 axis as a major contributor to the neuroprotective potential of PLG. In a primary cortical neuronal cell model, grape wine polyphenols prevent axonal apoptosis and act via mitochondrial SIRT3 activation in axons [61]. In addition to PLG, a variety of bioactive compounds in plants can regulate the expression of proteins related to mitochondrial dynamics to reduce mitochondrial abnormalities after exposure to neurotoxicants. For example, the decrease in mitochondrial membrane potential and mitochondrial DNA content in PC12 cells exposed to 6-OHDA can be improved by inhibiting DRP1 and Fis 1 proteins and inducing OPA1 protein expression with allicin in garlic [62]. Carnosic acid isolated from rosemary reverses 6-OHDA-induced mitochondrial dynamic disorders. It can enhance the expression of OPA1, prevent the release of cytochrome c, and inhibit the activation of apoptosis-related proteins [63].

Neuroinflammation is also an important pathological factor in C9-ALS [64]. Neuroinflammation may be an upstream cause of disease, and the sustained initiation of an inflammatory response resulting from neuronal damage may also be detrimental to the remaining neurons and exacerbate the disease process. Some reports suggest that PLG also has anti-inflammatory properties. It can inhibit NF-κB [33] and JAK2/STAT3 activity [47] and initiate Nrf2 and PPARα signaling pathways [32]. Therefore, in addition to improving mitochondrial function, PLG can also inhibit the inflammatory response to the neuronal microenvironment.

## 5. Conclusions

On the basis of the above, I established a C9-ALS drug screening model expressing PR-DPR in mouse NSC-34 motor neurons. In this model, I observed defects in mitochondrial dynamics caused by PR-DPR and apoptosis induced by ROS production. Using this model, I found that PLG promotes mitochondrial fusion and inhibits ROS production and apoptosis. Mechanistically, the neuroprotective effect of PLG is partly achieved by regulating SIRT3 activity and enhancing the deacetylation of OPA1. Therefore, PLG alleviates the toxicity of PR-DPR derived from C9-ALS, and its efficacy can be further evaluated using motor neurons derived from induced pluripotent stem cell lines from C9-ALS patients or transgenic mice in the future.

## Data Availability

All data used and analyzed during the current study are available from the author upon reasonable request.
